# Myelofibrosis predicts deep molecular response 4.5 in chronic myeloid leukaemia patients initially treated with imatinib: An extensive, multicenter and retrospective study to develop a prognostic model

**DOI:** 10.1002/ctm2.70101

**Published:** 2024-11-22

**Authors:** Tian Zeng, Xiudi Yang, Yi Wang, Dijiong Wu, Weiying Feng, Ying Lu, Xiaoqiong Zhu, Lirong Liu, Mei Zhou, Li Zhang, Yanping Shao, Honglan Qian, Feng Zhu, Yu Chen, Dan Cao, Li Huang, Xiaoning Feng, Lili Chen, Gang Zhang, Jing Le, Weiguo Zhu, Yongming Xia, Yanxia Han, Yongqing Jia, Guoyan Tian, Hui Zhou, Linjuan Xu, Xiufeng Yin, Qinli Tang, Yuefeng Zhang, Guoli Yao, Xianghua Lang, Kaifeng Zhang, Xiujie Zhou, Junbin Guo, Jinming Tu, Jianzhi Zhao, Gongqiang Wu, Huiqi Zhang, Xiao Wu, Qiulian Luo, Lihong Cao, Binbin Chu, Wei Jiang, Haiying Wu, Liansheng Huang, Meiwei Hu, Muqing He, Jingjing Zhu, Hongyan Tong, Jie Jin, Jian Huang

**Affiliations:** ^1^ Department of Hematology The First Affiliated Hospital Zhejiang University School of Medicine Hangzhou China; ^2^ Zhejiang Clinical Medical Research Center of Hematology Hangzhou China; ^3^ CML Cooperation Group of Zhejiang Hematology Zhejiang China; ^4^ Department of Hematology The First Affiliated Hospital of Ningbo University Ningbo China; ^5^ Department of Hematology The First Affiliated Hospital of Zhejiang Chinese Medical University Hangzhou China; ^6^ Department of Hematology Shaoxing People's Hospital Shaoxing China; ^7^ Department of Hematology The Affiliated People's Hospital of Ningbo University Ningbo China; ^8^ Department of Hematology The Fourth Affiliated Hospital Zhejiang University School of Medicine YiWu China; ^9^ Department of Hematology The Affiliated Hangzhou First People's Hospital Zhejiang University School of Medicine Hangzhou China; ^10^ Department of Hematology Zhuji Affiliated Hospital of Shaoxing University Shaoxing China; ^11^ Department of Hematology Taizhou Hospital of Zhejiang Province, Wenzhou Medical University Taizhou China; ^12^ Department of Hematology The First Affiliated Hospital of Wenzhou Medical University Wenzhou China; ^13^ Department of Hematology Zhoushan Hospital Zhejiang University School of Medicine Zhoushan China; ^14^ Department of Hematology College of Medicine Lishui Hospital Zhejiang University Lishui China; ^15^ Department of Hematology Huzhou Central Hospital Huzhou China; ^16^ Department of Hematology The Affiliated Jinhua Hospital of Wenzhou Medical University Jinhua China; ^17^ Department of Hematology Lishui People's Hospital Lishui China; ^18^ Department of Hematology The First People's Hospital of Taizhou Taizhou China; ^19^ Department of Hematology The First Hospital of Jiaxing The Affiliated Hospital of Jiaxing University Jiaxing China; ^20^ Department of Hematology Ningbo Medical Center Lihuili Hospital Ningbo China; ^21^ Department of Hematology Shaoxing Second Hospital Shaoxing China; ^22^ Department of Hematology The Affiliated Yangming Hospital of Ningbo University, Yuyao People's Hospital of Zhejiang Province Yuyao China; ^23^ Department of Hematology The Second Hospital of Jiaxing Jiaxing China; ^24^ Department of Hematology Jinhua Hospital Zhejiang University School of Medicine Jinhua China; ^25^ Department of Hematology The Affiliated Hospital of Hangzhou Normal University Hangzhou China; ^26^ Department of Hematology The Quzhou Affiliated Hospital of Wenzhou Medical University Quzhou People's Hospital Quzhou China; ^27^ Department of Hematology Sir Run Run Shaw Hospital Zhejiang University School of Medicine Hangzhou China; ^28^ Department of Hematology The First People's Hospital of PingHu PingHu China; ^29^ Department of Hematology First People's Hospital of Linping District Hangzhou China; ^30^ Department of Hematology Yongkang First People's Hospital Affiliated to Hangzhou Medical College Yongkang China; ^31^ Department of Hematology Xinchang County People's Hospital Xinchang China; ^32^ Department of Hematology Haining People's Hospital Haining China; ^33^ Department of Hematology The First People's Hospital Of Wenling Wenling China; ^34^ Department of Hematology The People's Hospital of Longyou City Longyou China; ^35^ Department of Hematology Shaoxing Central Hospital Shaoxing China; ^36^ Department of Hematology Dongyang Hospital Affiliated to Wenzhou Medical University Dongyang People's Hospital Dongyang China; ^37^ Department of Hematology The First People's Hospital of Huzhou Huzhou China; ^38^ Department of Hematology The Affiliated Hospital of Medical School Ningbo University Ningbo China; ^39^ Department of Hematology Yiwu Central Hospital Yiwu China; ^40^ Department of Hematology Shulan (Hangzhou) Hospital Affiliated to Zhejiang Shuren University Shulan International Medical College Hangzhou China; ^41^ Department of Hematology Oncology Ningbo Mingzhou Hospital Ningbo China; ^42^ Department of Hematology Shangyu People's Hospital of Shaoxing Shaoxing China; ^43^ Department of Hematology Tongde Hospital of Zhejiang Province Hangzhou China; ^44^ Department of Hematology The Second Affiliated Hospital of Zhejiang University School of Medicine Hangzhou China; ^45^ Department of Hematology The Second Affiliated Hospital of Zhejiang Chinese Medical University Hangzhou China; ^46^ Department of Hematology The Second Affiliated Hospital of Wenzhou Medical University Wenzhou China

Dear Editor,

Attaining a deep molecular response (DMR) has emerged as a desirable therapeutic target in chronic myeloid leukaemia (CML) patients considered for treatment‐free remission (TFR).^1^ Switching to second‐line therapy after failing to reach DMR with frontline imatinib has been recognized as an effective approach.^2^ The optimal timing for switching to more potent tyrosine kinase inhibitors (TKIs) to achieve timely DMR remains controversial.^3^ Myelofibrosis (MF) is associated with poor overall survival and a greater risk of disease progression in CML patients.^4^, ^5^, ^6^ However, the associations between MF and DMR in CML patients initially treated with imatinib have not been extensively studied, and we aimed to fill this gap.

Our study involved 925 CML patients with bone marrow biopsies who initially received imatinib from 1 January 2010 to 1 August 2022 (Figure S1). MF was evaluated by experienced pathologists through bone marrow biopsies and graded from 0 to 3 based on the WHO grading system (Table S1).^7^ In this study, patients with MF‐1 or higher were classified as having MF as a crucial complication of CML. The demographic and clinical characteristics of the enrolled patients, categorized by MR4.5 status, are depicted in Figure 1A. Different MF grades were significantly associated with both overall survival (log‐rank *p* = .015) and MR4.5‐free survival (log‐rank *p* < .001) (Figure S2). Patients who achieved MR4.5 had a significantly higher proportion of non‐MF cases (81.26% vs. 63.99%, *p* < .001) (Figure 1B). A correlation heatmap of different variables revealed that the white blood cell (WBC) count had a moderate, significant negative correlation with haemoglobin (HGB) levels (*r* = ‐0.58) (Figure 1C).

**FIGURE 1:**
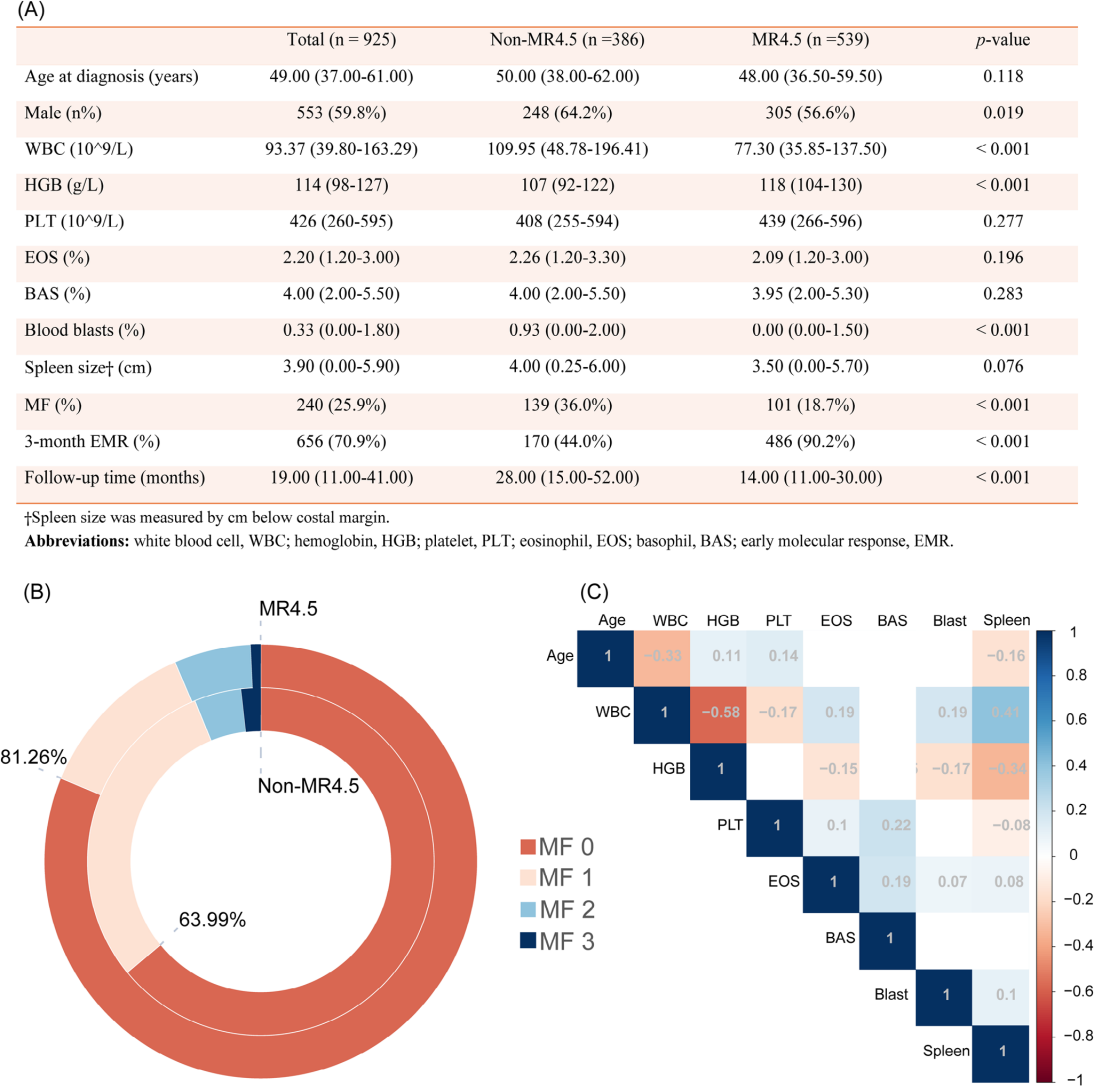
Baseline and clinical characteristics of the enrolled subjects. (A) Baseline demographic and clinical characteristics of the study cohort stratified by MR4.5 status. (B) Distribution of MF degrees across the MR4.5 and non‐MR4.5 groups. (C) A correlation heatmap. Categorical variables are expressed as frequencies and were compared using the χ2 test. Continuous variables are expressed as medians and interquartile ranges (IQRs) and were compared using the Mann‒Whitney U test. Spearman's correlation analysis was conducted to examine the relationships between continuous variables.

The 925 subjects were allocated to a training set and a validation set following a 7:3 ratio using a random splitting method via the ‘Sample’ function in R to ensure unbiased and random patient selection. No significant differences were found between the two datasets (Table S1). The Kaplan‐Meier (K‐M) curves revealed that patients with MF at diagnosis had a greater probability of remaining MR4.5‐free compared with those without MF (*p* < .001) (Figure 2A, a). Further analysis with a landmark at 18 months revealed that the inverse association was significant only after 18 months (*p* < .001) (Figure 2A, b). Considering that the intersection of two curves in the K‐M analysis might decrease the statistical efficiency, we concurrently plotted the restricted mean survival time (RMST) at 5 years (Figure 2B). The 5‐year RMST was 39.05 months in MF patients and 33.44 months in non‐MF patients.

**FIGURE 2:**
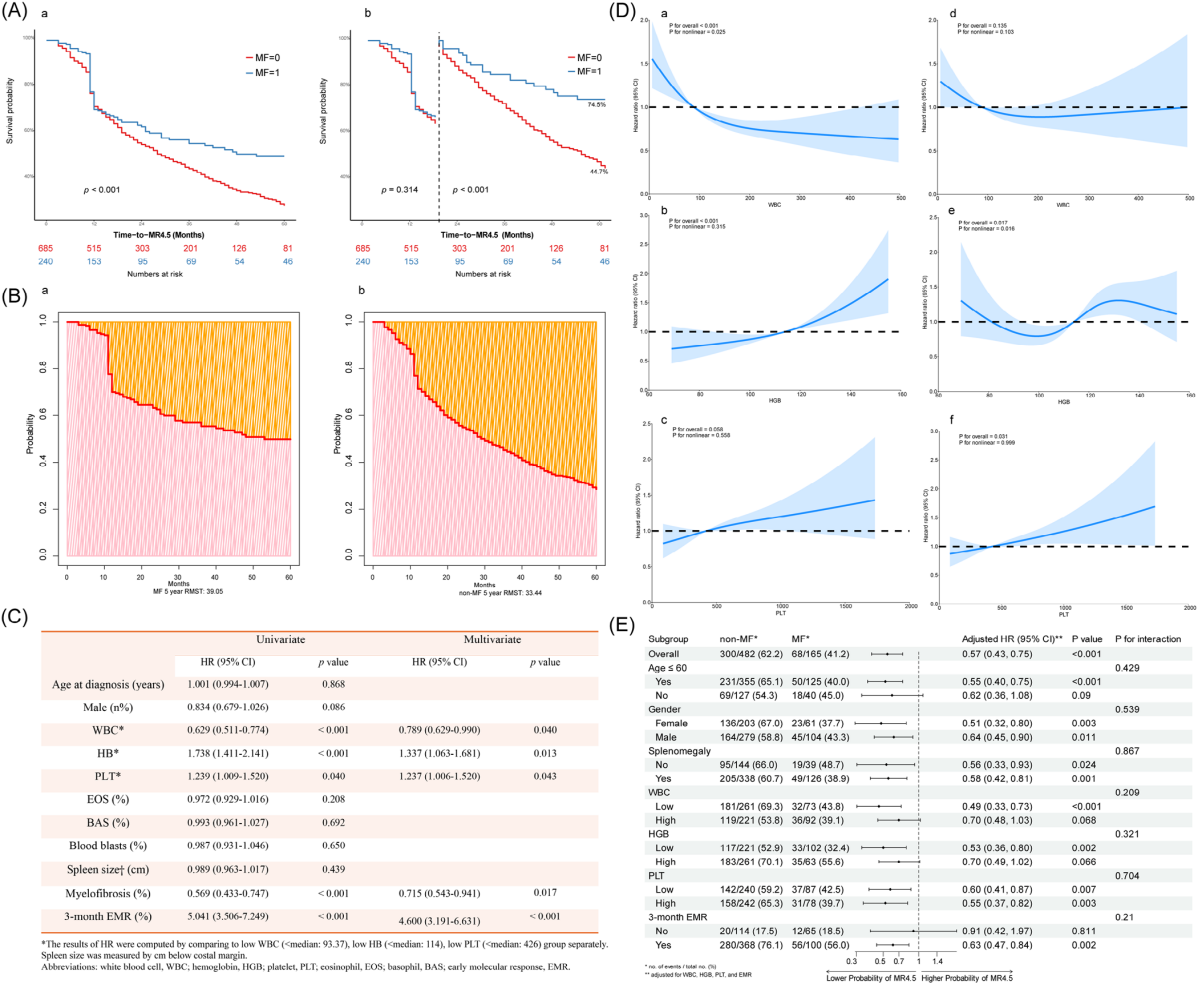
Survival analyses and subgroup analyses. (A) The Kaplan‐Meier (K‐M) curves and a landmark analysis at 18 months demonstrating MR4.5‐free survival differences between MF and non‐MF patients (a, b). (B) Restricted mean survival time (RMST) for MR4.5‐free survival differences between MF and non‐MF patients (a, b). (C) Univariate and multivariate Cox regression analyses of CML patients from the training cohort. (D) Univariate restricted cubic spline (RCS) models for white blood cell (WBC) (a), hemoglobulin (HGB) (b) and platelet (PLT) (c) and multivariate RCS for WBC (d), HGB (e) and PLT (f). (E) Subgroup analyses. The log‐rank test, landmark analysis and RMST were used to investigate survival. Significant factors were identified via univariate analysis (*p *< .05) and further analyzed via multivariate Cox analyses. The RCS models were fitted with 3 knots at the 10th, 50th and 90th percentiles of the WBC, HGB and PLT counts. Multivariate analyses were adjusted for EMR, MF, WBC, HGB and PLT.

In the training cohort, univariate Cox regression revealed that WBC, HGB, platelet (PLT), MF and 3‐month early molecular response (EMR) were risk factors for the incidence of MR4.5. After adjustments, these variables were found to be independent risk factors (Figure 2C). Specifically, an EMR was linked to a hazard ratio (HR) of 4.600 (95% confidence interval [CI]: 3.191–6.631), with a *p‐*value of < .001. In contrast, MF was linked to a 28.5% lower likelihood of achieving MR4.5 compared with non‐MF (HR: 0.715, 95% CI: 0.543–0.941, *p* = .017). Furthermore, restricted cubic spline (RCS) models indicated a significant dose‐response relationship of both WBC and HGB with MR4.5 (*p* for overall < .001) (Figure 2D, a,b). Intriguingly, after adjusting for confounding factors, an S‐shaped association between HGB and MR4.5 (*p* for overall = .017, *p* for nonlinear = .016) was observed (Figure 2D, f). Additionally, PLT presented a positive linear correlation (*p* for overall = .031, *p* for nonlinear = .999) with MR4.5 (Figure 2D, f).

Subgroup analyses were further performed to determine whether MF's predictive value for MR4.5 remained consistent across different demographic and clinical characteristics (Figure 2D). Analyses based on sex, splenomegaly and PLT revealed that MF was significantly negatively correlated with MR4.5 across all subgroups. After adjusting for WBC, HGB, PLT and 3‐month EMR, the subgroup analysis based on age revealed that an inverse association between MF and MR4.5 was statistically significant only among individuals aged ≤ 60 years (HR: 0.55, 95% CI: 0.40–0.75).

The independent predictors from the training cohort, including MF, WBC, HGB, PLT and 3‐month EMR, were used to construct a nomogram for predicting MR4.5‐free survival at 5 years, and an example of applying this nomogram to a given patient is provided in Figure 3A. Additionally, we stratified patients into two risk categories based on the total points derived from this nomogram. The cutoff value of 156 points for risk stratification was selected via the ‘Surv_cutpoint’ function of the ‘Survminer’ package in R, with those scoring < 156 categorized as high risk and those scoring ≥ 156 as low risk. The K‐M survival curve indicated a substantial difference in outcomes between the two risk groups (*p* < .001) (Figure 3A). Physicians should consider switching patients categorized as high risk to more potent 2nd TKIs rather than continuing imatinib therapy after 3 months of monitoring.

**FIGURE 3:**
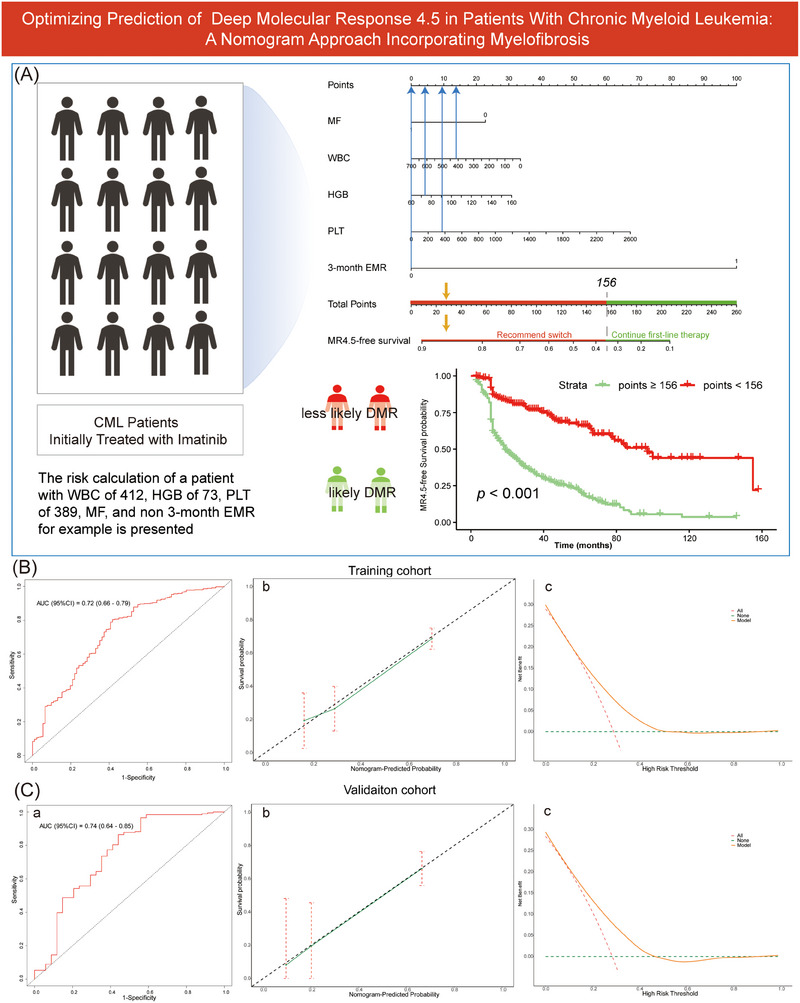
Development and validation of a nomogram for predicting 5‐year MR4.5‐free survival. (A) A nomogram for predicting 5‐year MR4.5‐free survival with an example and the risk stratification derived from it. (B) Validation in the training cohort. Receiver operating characteristic (ROC) curve (a). The calibration curve (b). Decision curve analysis (DCA) (c). (C) The validation from the validation cohort. ROC (a). The calibration curve (b). DCA (c). The nomogram was constructed based on data from the training cohort by including all the independent prognostic factors. Risk stratification based on the nomogram was performed via the ‘Surv‐cutpoint’ function in R. Calibration curves were constructed to evaluate the model's performance in terms of evaluating patient prognosis.

Our nomogram model exhibited a robust level of discriminative ability. In the training set, the area under the curve value was 0.72. Moreover, in the validation set, the value was 0.74. Furthermore, calibration curves demonstrated that MR4.5‐free survival estimates were aligned with the diagonal line. Additionally, the results of decision curve analyses demonstrated that the net benefits of applying our model surpassed those of overall interventions and no intervention approaches across most risk thresholds (Figure 3B,C). The validation of the nomogram for predicting MR4.5 at 1 year and 3 years is presented in the supplementary materials (Figures S3 and S4). The associations between the assessed variables, particularly EMR, and DMR have been reported in previous studies.^8^ However, this study uniquely integrates these variables along with MF into an intuitive nomogram model, offering a visual and accessible way to predict the achievement of DMR. To ensure the model's transportability and generalizability, further external validation in different populations is warranted.^9^

In conclusion, this study, concentrating on the endpoint of DMR, conducted the largest multicenter retrospective analysis of MF in CML to date. Additionally, to guide the treatment switch from imatinib to second‐line therapies, a visual, accessible and well‐validated model was developed to identify patients less likely to achieve DMR.

## AUTHOR CONTRIBUTIONS

Tian Zeng designed the framework of the letter and drafted the manuscript. Xiudi Yang, Yi Wang, Dijiong Wu, Weiying Feng, Ying Lu, Xiaoqiong Zhu, Lirong Liu, Mei Zhou, Li Zhang, Yanping Shao and Honglan Qian collected the data. Feng Zhu, Yu Chen, Dan Cao, Li Huang, Xiaoning Feng, Lili Chen, Gang Zhang, Jing Le, Weiguo Zhu and Yongming Xia performed patient follow‐ups. Yanxia Han, Yongqing Jia, Guoyan Tian, Hui Zhou, Linjuan Xu, Xiufeng Yin, Qinli Tang, Yuefeng Zhang, Guoli Yao, Xianghua Lang, Kaifeng Zhang and Xiujie Zhou performed data analysis. Junbin Guo, Jinming Tu, Jianzhi Zhao, Gongqiang Wu, Huiqi Zhang, Xiao Wu, Qiulian Luo, Lihong Cao, Binbin Chu and Wei Jiang generated figures and tables. Haiying Wu, Liansheng Huang, Meiwei Hu, Muqing He and Jingjing Zhu provided final modifications to the manuscript. Hongyan Tong, Jie Jin and Jian Huang conceived and supervised the study. All authors contributed to manuscript revisions and approved the final manuscript as submitted.

## CONFLICT OF INTEREST STATEMENT

The authors declare no conflict of interest.

## FUNDING INFORMATION

This research was funded by the Key R&D Program of Zhejiang (No. 2022C03137) and the Zhejiang Medical Association Clinical Medical Research Special Fund Project (No. 2022ZYC‐D09).

## ETHICS STATEMENT

This study was approved by the Ethics Committee of the First Affiliated Hospital, School of Medicine, Zhejiang University Institutional Review Board and was conducted in compliance with the Declaration of Helsinki. Written informed consent was waived due to the retrospective nature of the study and the use of anonymized data.

## Supporting information



Supporting Information

## Data Availability

The data that support the findings of this study are available on request from the corresponding author. The data are not publicly available due to privacy or ethical restrictions.
