# Supplementary material for: Myelofibrosis predicts deep molecular response 4.5 in chronic myeloid leukaemia patients initially treated with imatinib: An extensive, multicenter and retrospective study to develop a prognostic model
Source: Clin Transl Med. 2024 Nov 22;14(11):e70101. doi: 10.1002/ctm2.70101 (PMC11583811; doi:10.1002/ctm2.70101)
Supplement: Supplementary file 1 — Supporting Information [file CTM2-14-e70101-s001.docx]

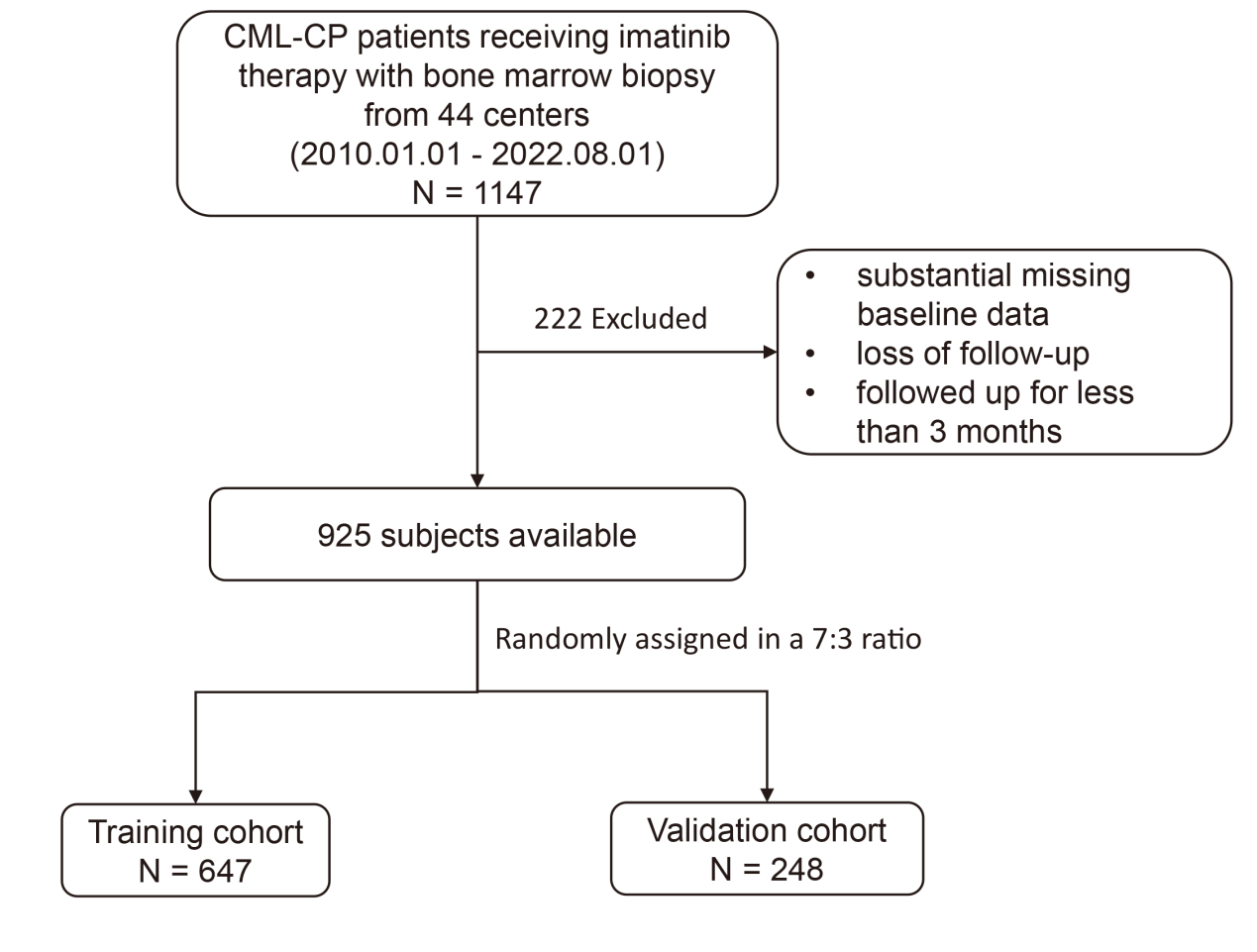


**Figure S1.** A flowchart of patients’ selection.


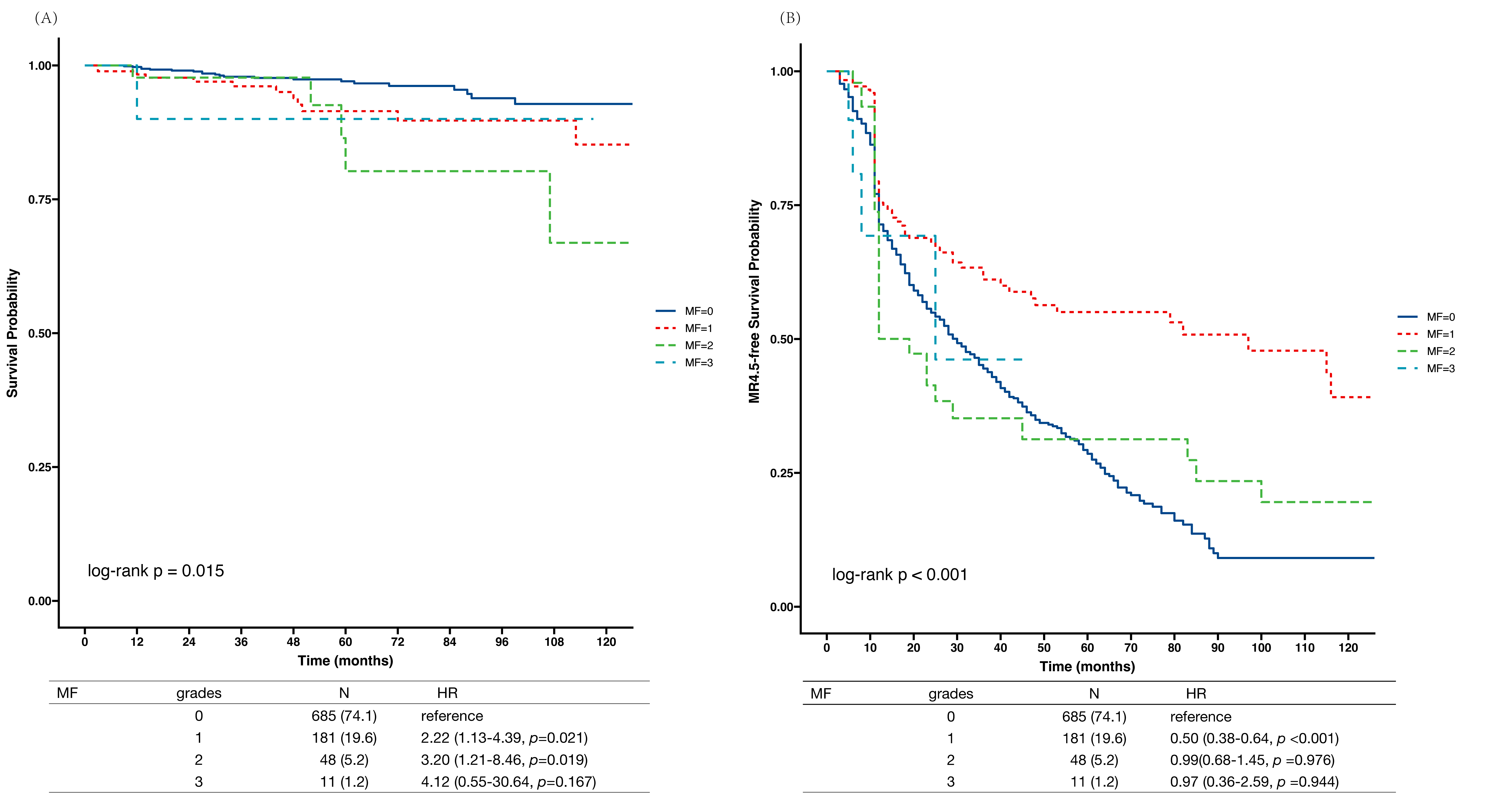


**Figure S2.** Overall survival (A) and MR4.5-free survival (B) of the patients according to MF grades.


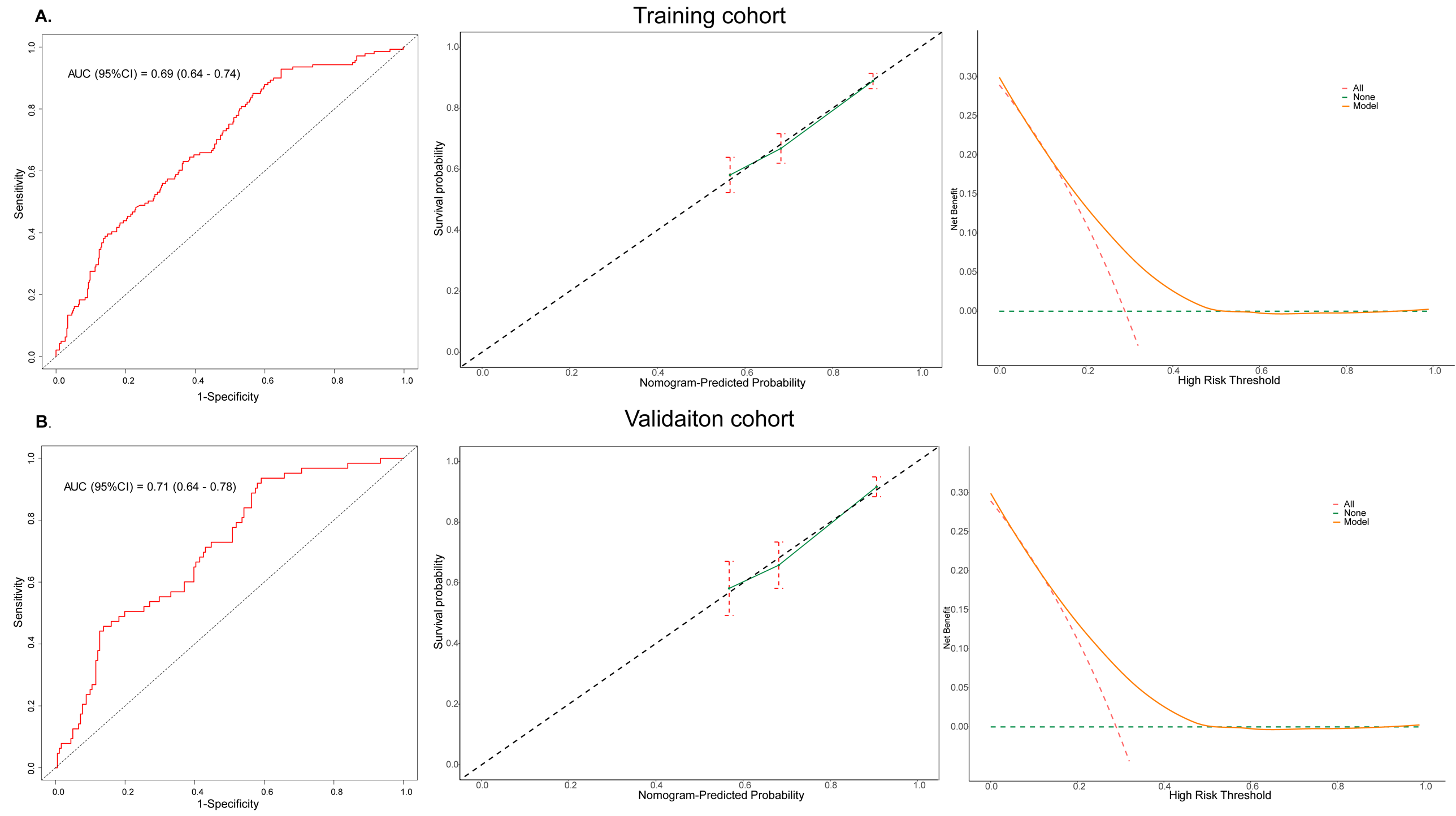


**Figure S3.** Evaluation of the performance of the nomogram model predicting 1-year MR4.5-free survival in the training (A) and validation (B) cohorts.


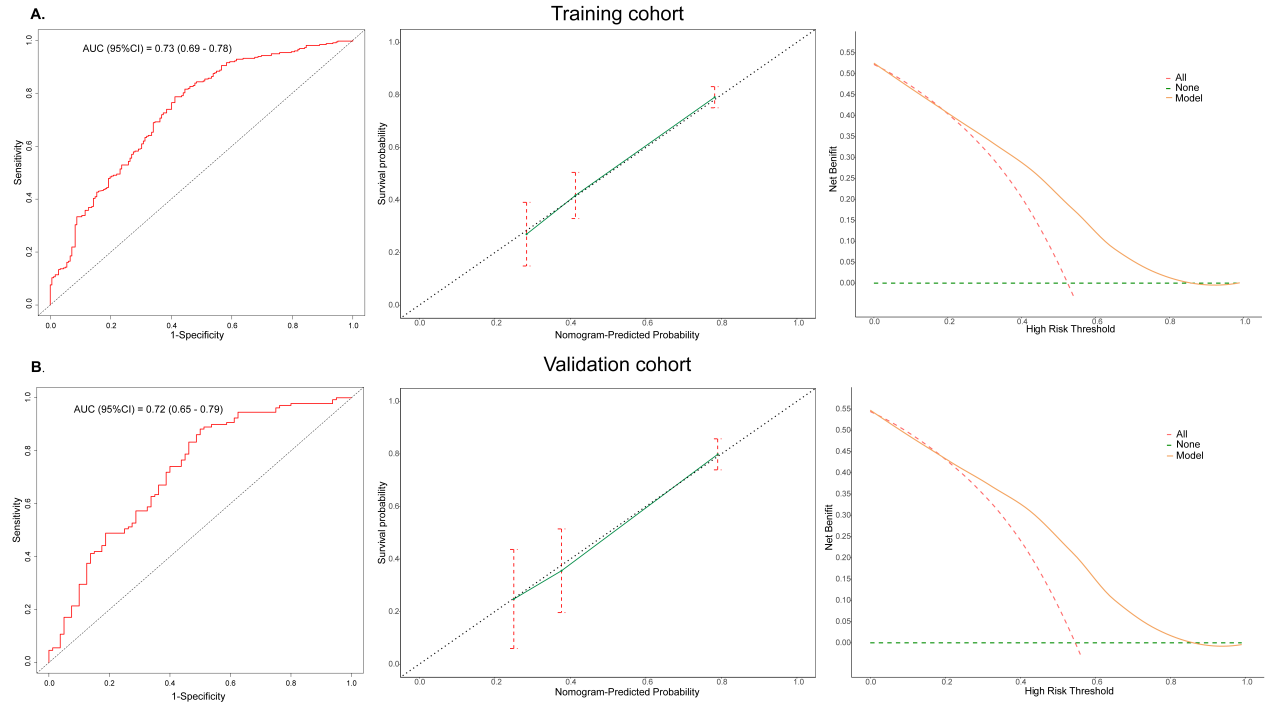


**Figure S4.** Evaluation of the performance of the nomogram model predicting 3-year MR4.5-free survival in the training (A) and validation (B) cohorts.

**Table S1.** Myelofibrosis grades according to the WHO grading system.

| **Myelofibrosis grades** | |
| --- | --- |
| MF-0 | Scattered linear reticulin with no intersections (crossovers) corresponding to normal BM |
| MF-1 | Loose network of reticulin with many intersections, especially in perivascular areas |
| MF-2 | Diffuse and dense increase in reticulin with extensive intersections, occasionally with focal bundles of thick fibers mostly consistent with collagen, and/or focal osteosclerosis |
| MF-3 | Diffuse and dense increase in reticulin with extensive intersections and coarse bundles of thick fibers consistent with collagen, usually associated with osteosclerosis |

**Table S2.** Baseline demographic and clinical characteristics in the training cohort and the validation cohort.

|  | **Validation (n = 278)** | **Training (n = 647)** | ***p*-value** |
| --- | --- | --- | --- |
| Age at diagnosis (years) | 47.50 (36.00-59.75) | 50.00 (38.00-61.00) | 0.138 |
| Male (n%) | 170 (61.15) | 383 (59.20) | 0.578 |
| WBC (10^9/L) | 100.00 (39.25-167.70) | 89.00 (39.80-159.75) | 0.663 |
| HB (g/L) | 114 (99-125) | 114 (98-128) | 0.983 |
| PLT (10^9/L) | 432 (257-592) | 424(263-596) | 0.972 |
| EOS (%) | 2.20 (1.20-3.00) | 2.17 (1.20-3.00) | 0.425 |
| BAS (%) | 3.77 (2.00-5.00) | 4.00 (2.00-5.68) | 0.115 |
| Blood blasts (%) | 0.00 (0.00-1.71) | 0.40 (0.00-1.86) | 0.457 |
| Spleen size† (cm) | 3.60 (0.00-5.70) | 3.90 (0.00-6.00) | 0.842 |
| Myelofibrosis (%) | 75 (26.98) | 165 (25.50) | 0.639 |
| 3-month EMR (%) | 188 (67.63) | 468 (72.33) | 0.148 |
| Follow-up time (months) | 20.00 (11.00-41.00) | 19.00 (11.00-42.00) | 0.765 |

†Spleen size was measured by cm below costal margin.

**Abbreviations:** white blood cell, WBC; hemoglobin, HGB; platelet, PLT; eosinophil, EOS; basophil, BAS; early molecular response, EMR.
